# Non-diagnostic based approaches to helping children who could be labelled ADHD and their families

**DOI:** 10.1080/17482631.2017.1298270

**Published:** 2017-05-22

**Authors:** Sami Timimi

**Affiliations:** ^a^ Faculty of Health and Social Sciences, University of Lincoln, Lincoln, UK

**Keywords:** ADHD, diagnosis, labelling, treatment, critique

## Abstract

Mental health services are not always good for you. There are some troubling facts to confront such as the increase in the use of diagnostic based approaches and psychotropic medications for children and young people being associated with poorer rather than better outcomes. In this article I will outline some of the evidence around outcome as a result of treatment for young people diagnosed with attention deficit hyperactivity disorder (ADHD) and for those who are prescribed long-term stimulants. I will then discuss clinical approaches that move beyond a focus on symptom management that diagnostic paradigms encourage. This includes clinical models that take account of the diversity of contextual and relational issues that young patients present with and the possibility afforded of engaging in more positive and hopeful therapeutic approaches such as the Relational Awareness Programme (RAP).

Mental health services aren’t always good for you. Although over-diagnosis and unnecessary care is recognized as a growing problem across medicine (Macdonald & Loder, 2015), prognosis for most conditions dealt with by the rest of medicine has improved, often reflecting genuine technical advances, no such progress has been seen in the prognosis of those who use real-world mental health services in Western nations. Lambert (2010) concluded that 75% of people entering community mental health centres in the USA are either not responding to treatment, or deteriorating whilst in care. Hansen and colleagues (2002) reported a picture of routine clinical care in US mental health services in which only 20% of patients improved. In Britain, the Centre for Social Justice (2012) found that only 15% of people referred to a National Health Service mental health treatment project were achieving “recovery” by the time they left. In Australia, despite massive investment in mental health services in the past two decades, no corresponding improvement in the adult mental health of the population has been found (Jorm & Reavley, 2012).

A similar picture of poor outcomes in real-world child and adolescent mental health services (CAMHS) is also found. Weisz and colleagues (1995) reported that the overall effect size of change for those attending community CAMHS, compared to those who were not, was close to zero, a finding replicated in further studies (Weiss, Catron, & Harris, 1999, 2000). Jörg and colleagues (2012) also found that, across a variety of measures, those who continuously attended community CAMHS had higher levels of self-rated distress/problems when compared to a matched community sample with similar levels of initial distress/problems, but who did not to access CAMHS. Kazdin (2004) reported that 40–60% of youth who begin treatment drop out against advice. Warren and colleagues (2009, 2010) found a deterioration rate of 24% amongst children in public CAMHS settings.

Whitaker (2010) has documented a tripling of the number of people categorized as disabled mentally ill in the USA over the past two decades. Similarly, the numbers of youth in America categorized as having a disability because of a mental condition leapt from around 16,000 in 1987 to 560,000 in 2007. Mental disorders have also become the most common reason for receiving benefits in the UK, with the number of claimants rising by 103% from 1995 to 2014. Claimants with other conditions fell by 35% (Viola & Moncrieff, 2016).

Of particular concern is the increase in the rate of ADHD diagnosis and stimulant prescription to young people. In the USA it is estimated that 11% of children aged between 4 and 17 have been diagnosed with ADHD and between half and two-thirds of these are then prescribed stimulants (Sharpe, 2014). In the UK prescribing of stimulants rose from about 6000 prescriptions a year in 1994 to over 1 million by 2013; a staggering 17,000% rise in two decades (Timimi, 2015).

## ADHD is a fact of culture, not a fact of nature

It requires little intellectual effort to reach the inescapable conclusion that the concept and definition of ADHD is replete with problems around reliability and validity. For example, diagnostic guidelines note that ADHD behaviours may be minimal or absent in a number of settings such as when the person is under close supervision, a novel setting, or engaged in especially interesting (to them) activities (Whitely, 2015). Even if a genetic basis for certain behavioural characteristics were found (and we are a long way from that) we still have to run the “so what” test and ask why such behaviours should be treated as disorders rather than differences. Deciding where to draw the line between what we consider part of the “ordinary” spectrum of behaviours and what we decide is “pathological” is more dependent on cultural than scientific processes. I have previously argued (Timimi, 2005; Timimi & Leo, 2009) that the rapid expansion in the use of culturally constructed diagnoses like ADHD, together with giving children powerful stimulant medications to control their behaviour, is more a damning indictment of the position of children in neo-liberal cultures, than an indication of scientific progress. The continuing absence of biological support, together with the avoidance of engagement with questions of validity, leads me to believe that this assertion remains relevant.

In psychiatry (apart from some forms of dementia and a few other known organically based conditions), there is no such thing as diagnosis. In medicine diagnosis is the process of determining which disease or condition *explains* a person’s symptoms and signs. Diagnosis therefore points to causal processes. Making an accurate diagnosis is a technical skill that enables effective matching of treatment to address a specific pathological process. Pseudo-diagnoses like ADHD cannot explain behaviours as there are only “symptoms” that are descriptions (not explanations) of behaviours. Even using the word “symptom” may be problematic, as in medicine “symptoms” usually refers to patients’ suffering/experience as a result of an underlying disease process and is therefore associated in our minds with a medical procedure leading to an explanation for the “symptom”. Consider the following example: If I were to ask the question “what is ADHD?” then it is not possible for me to answer that question by reference to a particular known pathological abnormality. Instead I will have to provide a description, such as ADHD is the presence of the behaviours of hyperactivity, impulsivity, and poor attention (plus a few extra qualifiers such as age of onset). Contrast this with asking the question “what is diabetes?” If I were to answer this question in the same manner by just describing symptoms such as needing to urinate excessively, thirst and fatigue, I could be in deep trouble as a medical practitioner as there are plenty of other conditions that may initially present with these symptoms and diabetes itself may not present with these symptoms in a recognizable way. In order to answer the question “what is diabetes?” I have to refer to its pathology involving abnormalities of sugar metabolism. I would then get independent (to my subjective opinion) empirical data to support or otherwise my hypothesis about what may be “causing” the patient’s described experiences (such as testing the urine and/or blood for levels of glucose). In the rest of medicine therefore, my diagnosis explains and has some causal connection with the behaviours/symptoms that are described. Diagnosis in that context sits in a “technical” explanatory framework. In psychiatry what we are calling diagnosis (such as ADHD) will only describe but is unable to explain.

The problem of using a classification like ADHD to explain an observed set of behaviours (i.e. as a diagnosis) can be illustrated by asking another set of questions. If I was to ask “why” a particular child cannot concentrate, is hyperactive and shows impulsivity and I were to answer that these behaviours are *caused* by ADHD, then a legitimate question to ask is “how do you know that they are caused by ADHD?” The only answer I can give to that question is that I know it’s ADHD because the child is presenting with hyperactivity, impulsivity and poor attention. In other words if we try to use a classification that can only describe in order to explain, we end up with what philosophically is known as a “tautology”. It is troubling when doctors use a diagnosis like ADHD to explain and cannot see this problem of tautological circularity. Furthermore, the idea that a diagnosis like ADHD “explains” behaviours, risks undermining our ability to attend to a whole host of other real-life factors that may have an important role to play in the development of ADHD behaviours in some children; from lack of sleep to witnessing domestic violence, from being the youngest in the classroom to struggling to keep up with the academic demands of school and so on.

This means that in psychiatry we are mostly working with a system for classification that is descriptive, *but not diagnostic*. As a classification it can have its uses such as recognizing and validating people’s struggles, as well as administrative and communication functions; but only if such a system of classification can be shown to have other advantages (such as with clinical outcomes).

As ADHD is not a medical diagnosis, but a descriptive classification, we have no empirical method for defining “caseness”. The definition of what qualifies as a case is thus arbitrary and depends on the standards employed by the diagnoser, influenced by whatever the prevailing ideology concerning diagnosis they have been exposed to. As a result we cannot eliminate wide variation in “diagnostic” practice or come to any valid conclusion about what percentage of the population could be considered as “over” or “under” diagnosed.

As ADHD is not a medical diagnosis it is not surprising that there has been a failure to find any specific and/or characteristic biological abnormality such as characteristic neuroanatomical, genetic or neurotransmitter abnormalities (Campo et al., 2013; Timimi & Timimi, 2015; Whitely, 2015).

## Does the concept of ADHD help improve clinical outcomes?

The evidence on outcomes in mental health in general finds that the process of matching treatment models to a diagnosis results in virtually no clinically significant impact on outcomes (Timimi, Tetley, Burgoine, & Walker, 2013), a finding that extends to ADHD (Miller, Wampold, & Varhely, 2008). Instead of specific factors associated with specific therapeutic modalities, it seems “common factors” (factors that all therapeutic endeavours have in common) are the biggest contributors to variance in outcome from treatment. With regards to what “common factors” are particularly influential in the likelihood of a positive outcome (or not), it seems that it is factors outside of therapy (such as socio-economic status, patient expectation, the availability of social support, etc.) that have the largest impact on outcomes and recovery rates. Within treatment, the factor that has the greatest impact on outcomes is the therapeutic alliance (as rated by the patient), with matching treatment model to diagnosis having an insignificant impact (Duncan, Miller, Wampold, & Hubble, 2010; Wampold, 2001). This relationship between the alliance and outcome seems remarkably robust across treatment modalities and clinical presentations (Castonguay & Beutler, 2005). Furthermore, many of the “technologies” (such as specific psychotherapy models) have been developed in a Western cultural context and researched in predominantly Western societies, raising questions about their suitability when working with communities who do not share similar beliefs and practices.

Most of the controversy in ADHD treatments revolves around the use of stimulant medication. The evidence does not favour the continual increases in stimulant prescribing that has been occurring in most Western societies. Reviews of pharmacotherapy studies note the inadequate reporting of drug trial methodology, publication bias, limited reliability of results, inadequate data regarding adverse events, and lack of evidence of long term benefit (e.g. Storebø et al., 2015). The most commonly cited reference in support of using stimulant medication is the American multi-modal treatment of ADHD (MTA) study, which concluded, in a 14-month randomized controlled trial, that patients receiving medication had better outcomes compared to those who did not (MTA Cooperative Group, 1999). The MTA study compared outcomes from four treatment groups: medication (stimulants) only, behaviour therapy only, combined medication and behaviour therapy, and routine community care. The authors concluded that after 14 months of treatment, there was more reduction of ADHD symptoms in the medication only and combined treatment groups than the behavioural therapy only group, who in turn were better than the routine community care group. However, two thirds of the routine community care group were also on the same medication as the medication arm of the study, yet had the poorest outcomes. Furthermore, the behavioural treatment arm consisted of an intensive course that was completed during the 14 months, so that by the time of the 14-month evaluation, the families receiving the behavioural therapy intervention had completed it up to 9 months before the assessments, whilst the medication arm included appointments right up to 14 months. These two points raise the distinct possibility of placebo being the reason for better outcomes in the medication and combined treatment arms at 14 months (Pelham, 1999).

Follow-up at 3 years of the MTA study patients (now at ages 10–13 years with a mean age of 11.9 years), could not find support for continuing superiority of medication regardless of initial severity (Jensen et al., 2007). Additionally, those who used more medication during the 3 years were more likely to experience a deterioration in ADHD symptoms, had higher rates of delinquency, and were significantly shorter (by an average of 4 cm) and lighter (by 3 kg) than those who had not taken medication (Molina et al., 2007; Swanson et al., 2007). Naturalistic long term (3 years or more after starting treatment) follow-up studies have failed to demonstrate that long term use of stimulants is associated with any improved outcomes when you compare children diagnosed with ADHD who regularly take stimulants to those who do not. Where there are differences between the two groups (of those who take stimulants long term and those who do not) it is often found that it is the children on stimulants who have worse outcomes than those who have not taken them (regardless of initial severity) (e.g. Currie, Stabile, & Jones, 2013; Government of Western Australia, Department of Health, 2010; Langley et al., 2010; Riddle et al., 2012).

## Treating ADHD as a social construct

Diagnoses like ADHD have become popularized, in most Western countries, where the public have had exposure to the discourse of ADHD as a biological disorder to be treated with effective medications (Timimi & Timimi, 2015). As a result, our social construction of what is “normal” for children and for their parents has, for a few decades now, become narrowed. This greater problematization of children and their behaviour means that we are increasingly training all of us (parents, teachers, doctors, etc.) to focus our energies on “what is wrong”. Because our current definitions construct problems through lists of (negative) behaviours (e.g. “doesn’t do as told”, “doesn’t sit still”, etc.) what often goes missing in our evaluations are the emotional state and the more positive aspects of the child’s behaviour and/or intentions. What can then happen in treatment is a focus on techniques to control behaviour—in other words focusing on reducing the amount of “symptoms”—leaving behind an understanding of children at the emotional level, of their connection to the people and contexts around them, and of their resilience, competencies and strengths. Understanding that ADHD is a social construct provides a non-pathologizing starting point for helping children and their families.

Our ideas about treatment are just as socially constructed as diagnoses. The “common factors” evidence-based literature demonstrates that specific techniques are not as important as contextual factors and the human relationship in therapy. The technical aspect of care (matching treatment to diagnosis) has little impact on outcomes, whereas relational and contextual aspects of care are crucial (Bracken et al., 2012; Dunacn et al., 2010). Below are a few ideas that I and colleagues use and that start from a “social construction” assumption and use a context and relationship rich perspective. Whilst they may be viewed as “technical” (in that they have theory and technique), they employ the flexibility afforded by not focusing on matching treatment to any diagnosis, but trying techniques that may engage a particular young person or family. If people don’t respond positively we have open discussions about what other ideas might be worth trying.

## Relational Awareness Programme (RAP)

The RAP approach was inspired by the work of Howard Glasser (see for example, Glasser & Easley, 2007) who developed the “Nurtured Heart Approach” (NHA), which is a non-diagnostic, skills based approach, for parents of “intense” children who may present with challenging behaviours. RAP is an extension of the NHA approach, incorporating ideas from systemic and family therapy, and has also been developed into a parent group workshop programme and an e-learning format that supports it. The approach involves prioritizing building relationships over controlling behaviour (or symptoms). One of the problems with focusing on controlling of behaviour is that attempts to control behaviour can lead to further strain in the relationship, in a reinforcing negative cycle, where the more you try to control behaviour, the worse the relationship becomes, and the more the young person feels alienated or ostracized, the more they may demonstrate further aggravating behaviours. By focusing on relationships to start with we hope to build a better foundation for some mutual appreciation, before thinking about using boundaries and consequences.

We use the idea of “Emotion WARS” to help parents understand how this emotional dynamic may function. We are aware we are offering parents a framework to think about relationships, rather than a “truth” and this is regularly explained to participants in the group to allow for participants’ own knowledges, skills, insights, and creativity to be shared and reinforced:

### “W” is for wrongs

The first assumption is that we are programmed to notice more what’s going wrong than what’s going right. Our evolutionary survival was related to our ability to scan our environment for signs of danger and respond to these. As this is an instinctual aspect, it means that the more stressed we feel, the more likely we are to activate this instinct to try to fix immediately any perceived problem. Becoming preoccupied with what is going wrong means that whatever the cause of increased stress becomes irrelevant compared to the actual experience of stress. So whether it’s difficult finances, problems in marital relationship, the stress of school regularly ringing up, and so on, once we experience increased stress, we are more likely to end up focusing on what’s wrong, including with our children.

### “A” is for attachment

The next basic assumption is that children are born as essentially “emotion seeking” creatures. Attachments are created through our emotional relationships and what we seek is emotional energy from those we are attached to. In an infant’s world, there is no such thing as good or bad emotion. For the infant and growing child there is an instinctual drive towards seeking emotion so that any emotion (be that affection or anger) that comes back to them from a caregiver is experienced as rewarding in some way. Seeking emotional energy is a better way of describing what children do than seeking attention.

### “R” is for relationships

Relationship dynamics are then built up through these emotional energy ties that family members have to each other and over time this becomes a dynamic between any group of people who spend extended lengths of time together.

### “S” is for scripts

Over time we each develop roles within any group and these roles tend not to be ones that we have consciously chosen but rather the emotional, relational dynamic “settling” into a certain recognizable pattern that everybody in the group (often unconsciously) recognizes. For example, it is a well-known phenomenon that if you have a partner, one of you will end up being the one preoccupied with tidying up, another one may be preoccupied with sorting out the bills or getting children ready in the morning, and so on. Many then find that even if you want the roles to change, it doesn’t feel “right” when you do. So, for example, if you are the one who is concerned about keeping the house tidy and you yearn for your partner to also do this, when they do it, somehow they never seem to be able to do it in the “right” way and so you have to re-do it to what you consider to be the correct standard. This is a bit like each family member having a “script” that they follow which identifies their role and should any member come along with a new script that changes their usual role, it throws everybody else out because it doesn’t seem “natural”. There is thus a powerful and usually unrecognized emotional force from other members pushing anybody who goes “off script” back towards the script that everybody recognizes. So if a child occupies the role of say “the troublemaker”, family members will often assume that this child is in some way involved whenever trouble is happening.

### Changing the emotional dynamic

The concept of “Emotion WARS” then forms the background starting point in moving toward changing the emotional dynamics in the “relationship script” by helping parents, teachers and others in caring roles focus on relationship building by moving away from emotional energy reinforcing negatives and toward reinforcing positives. They then learn skills for dealing with issues such as rules, consequences, and their own emotional resilience. For our RAP group workshop model we have used a programme consisting of four 2½-h groups spread out over 2 months between the first and last groups. We have developed an e-learning platform which contains all the resources, including the PowerPoint slides, narrations, exercises and detailed advice on setting up and running the group to enable other practitioners to develop and run groups in their locality. Parents and carers of children with “challenging” behaviour (regardless of diagnosis) are invited. In November 2014 we conducted an evaluation of the outcomes from these groups, as we had outcome data from using the Outcome Rating Scale (ORS, Miller, Duncan, Brown, Sparks, & Claud, 2003) through asking the group participants to complete this scale at the start of each group. The ORS is filled in by the parent/carer according to their perception of how they think their child is feeling and functioning at home and out of home (such as with school and friendships).

We found that 80% of those who attended more than one workshop (57 out of 71; nine attended one session only and therefore were not included in this outcome figure), rated a clinically significant improvement and/or rated their child as being in the clinically non-significant range, by the last group. These figures should of course be viewed with caution as this was not part of a research project and therefore there was no control group. However, they offer some promise as many attendees reported going for multiple other interventions with their children prior to trying this group and the findings compare favourably with the real world setting outcomes found in the research quoted earlier. One of the most common feedback comments from participants who attended with a successful outcome is that what changed most for them was their “attitude” to their child; which became more understanding and co-operative.

## Other helpful ideas

RAP is a relationally based intervention. In some senses this mirrors what the evidence discussed earlier in this article finds, which is that therapy essentially takes place in the negotiated space of meanings that makes up the alliance. Therefore, I believe, we should be careful not to become too reliant on one set of ideas or method. What matches the rhythms of significance and meaning for each family may be different. As a result it’s useful to have a whole variety of ideas to draw from. Thus, possible therapeutic strategies are as numerous as there are people and families wanting help. As the ADHD label is unable to offer much more than a vague description, the specifics of each case differ; meaning that finding helpful therapeutic strategies may similarly differ depending on the family (for a fuller discussion of this please see Timimi, 2005, 2009; Timimi et al., 2013). The following ideas may prove helpful for some:

### Diet and nutrition

Try eliminating potential irritants (such as artificial additives), adding a daily multi-vitamin and mineral supplement and an EPA rich essential fatty acid, and balance the diet by removing excess sugars and dairy products.

### Fresh air and exercise

Enable your children to get plenty of opportunities for exercise (particularly outdoors) including chances for unstructured and unsupervised active play.

### Clear and consistent consequences

Notice your child’s strengths and support and encourage these in a positive direction. Use clear and consistent consequences for unwanted behaviour and work hard to stick to these and not give in (stay firm, keep calm). Try not to get drawn into arguments, which often feed negativity to everyone.

### Regular positive family time

Find opportunities to do things together as a family on a regular basis. Like all relationships we have to continue “working” on our relationships with our children (year after year after year after …).

### Communication and understanding

Talk to each other, but more importantly listen to each other (not the same thing). Try and understand your child’s point of view and help them understand yours. Create regular opportunities for you to communicate, listen and try and understand what’s on each of your minds.

The following “pitfalls”, leading to therapeutic strategies failing, are also worth keeping in mind:

### Giving up too quickly

With some interventions, unwanted behaviours can get worse before they start to improve, resulting in parents giving up on the strategy prematurely.

### Becoming hopeless following a setback

Setbacks are an inevitable part of any recovery process. Hopelessness can creep in and with it a sense of failure and a loss of confidence in the ability to bring about lasting change. When the inevitable setback occurs, remember it’s very common and don’t give up at this point.

### Unrealistic expectations

If we have unrealistic expectations of our children, then we will feel disappointed with them no matter what changes.

### Inconsistency

Children are often clever enough to spot opportunities that arise from inconsistencies in order to further their own desires, for example, by playing one parent off another.

### Unresolved difficulties between parents

This is where issues such as inconsistency can become a potentially serious obstacle to progress. In a situation where the parental couple have separated, it is vital for parents to put any continuing animosity towards one another to one side and keep the child out of any arguments.

### Unresolved issues from a parent’s own childhood

For example, if a parent had an unhappy relationship with their own parents resulting in them feeling hate or fear towards that parent, they may act with their own children in a way that is designed to avoid this happening to them, resulting in trouble enforcing boundaries with their own children.

### The anger-guilt-reparation cycle

In this “drama” a parent becomes infuriated with the child’s behaviour, imposes some sort of punishment (anger), they then calm down and feel that their punishment was unduly harsh (guilt), as a result they try to repair some of the damage they feel they have done and so may give some sort of treat or comfort to their child (reparation). The child may learn that any consequences imposed may be withdrawn and, indeed, may be followed by some sort of reward.

### Creation of a “safe zone”

We are exposed to constant messages telling us how dangerous the outside world is, particularly for children. The subsequent desire to protect our children from the perceived dangers of the world may hamper children’s capacity to develop the resilience they may need to cope in later life.

### Fear of change

This can be a change in any member of the family (see above about family scripts). Change usually causes a certain amount of anxiety and fear of the unknown, not just for the individual but also other family members.

### Lack of support

As the old African saying reminds us “it takes a village to raise a child”. Raising children demands a lot from parents both physically and mentally, and given the pressures they face, they need trusted partners, friends and other family members to provide emotional and practical support.

### Lack of time

Another feature of modern life is how busy and time stretched we are. With so many things to do and such little time to do them we may feel stressed and this often impacts on children.

### Unresolved trauma

Everybody reacts differently to trauma (be this abuse, loss of a parent, being assaulted, involved in a serious accident, etc.). For some, trauma may lead them to feel preoccupied, constantly worrying about their safety and the safety of others and they may present with poor concentration, as things like school work will be of limited importance to them. Others may have developed the psychological defence of “being on the go”; by constantly doing things it keeps their minds away from thinking about what has happened and what may happen in the future.

## Conclusion

ADHD is a cultural construct. It is often argued that the use of categorical constructs like ADHD enables the study of aetiology, treatment and prognosis. Evidence outlined above demonstrates that far from enabling any advancement of knowledge or clinical practice, it has created an illusion of progress and resulted in exposure of possibly millions of children and young people to unnecessary and potentially harmful medications. It has spurred on liberal use of stimulant medication, despite the lack of evidence for improved long term outcomes resulting from this. ADHD is an example of the “MacDonaldization” of children’s mental health where marketing and commodification of our anxieties about failure (as parents, teachers and individuals) has triumphed over science and good ethics (Timimi, 2017). The concept is well past its use-by date and should be discarded. Plenty of alternative therapeutic strategies exist and I have provided a brief description of some in this article. Sadly or gladly (depending on your perspective), in the real world children’s behaviour does not emerge out of predictable algorithms that enable us to accurately identify separate features caused by genes, parents, teachers etc., which then allows us to choose the “correct” treatment. Personally, I think it’s wonderful that the uniqueness of the children and families I see in practice challenges me to take the time to understand their specific worries, health problems, goals, dreams and talents, in a way that makes formulaic guidelines seem, not only redundant, but a hindrance to enacting the standards of good medical practice expected of us. If child psychiatry has anything to teach the rest of medicine, it is surely this—that for many presentations there is no short cut to understanding the whole person through their unique histories and context—good outcomes often depend on this.
